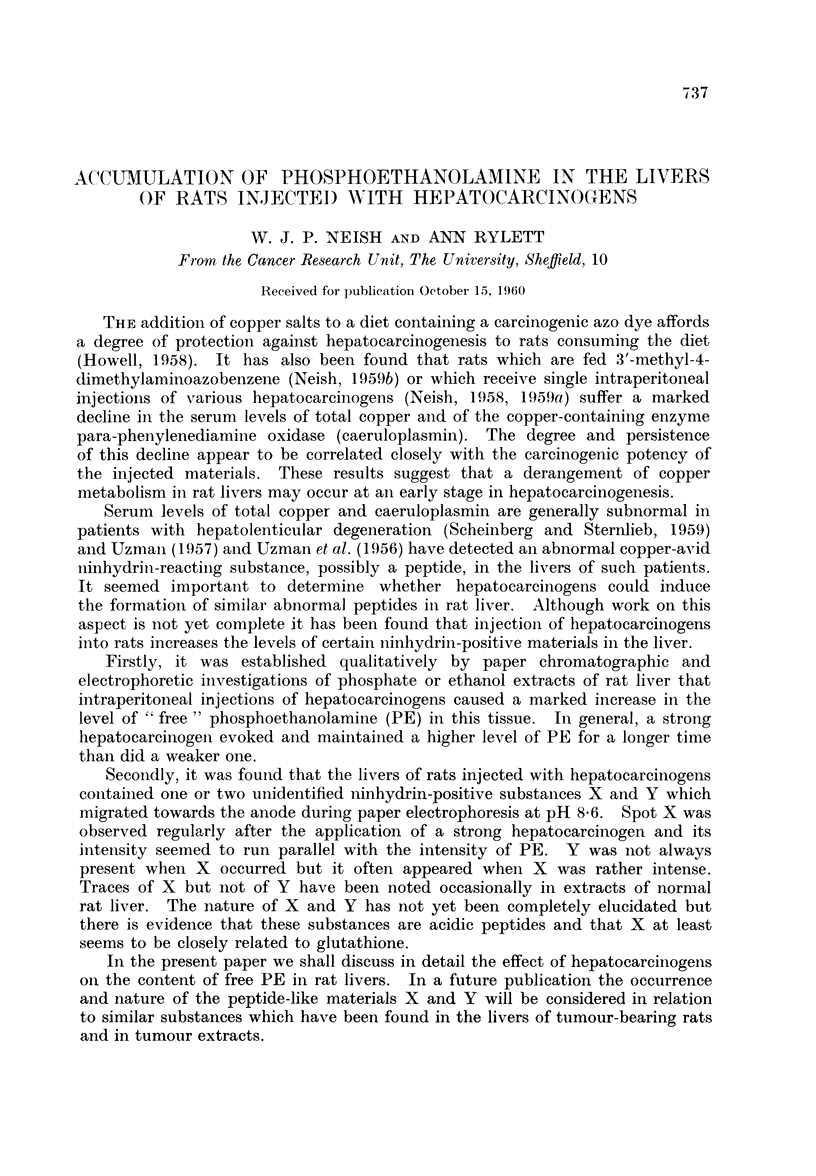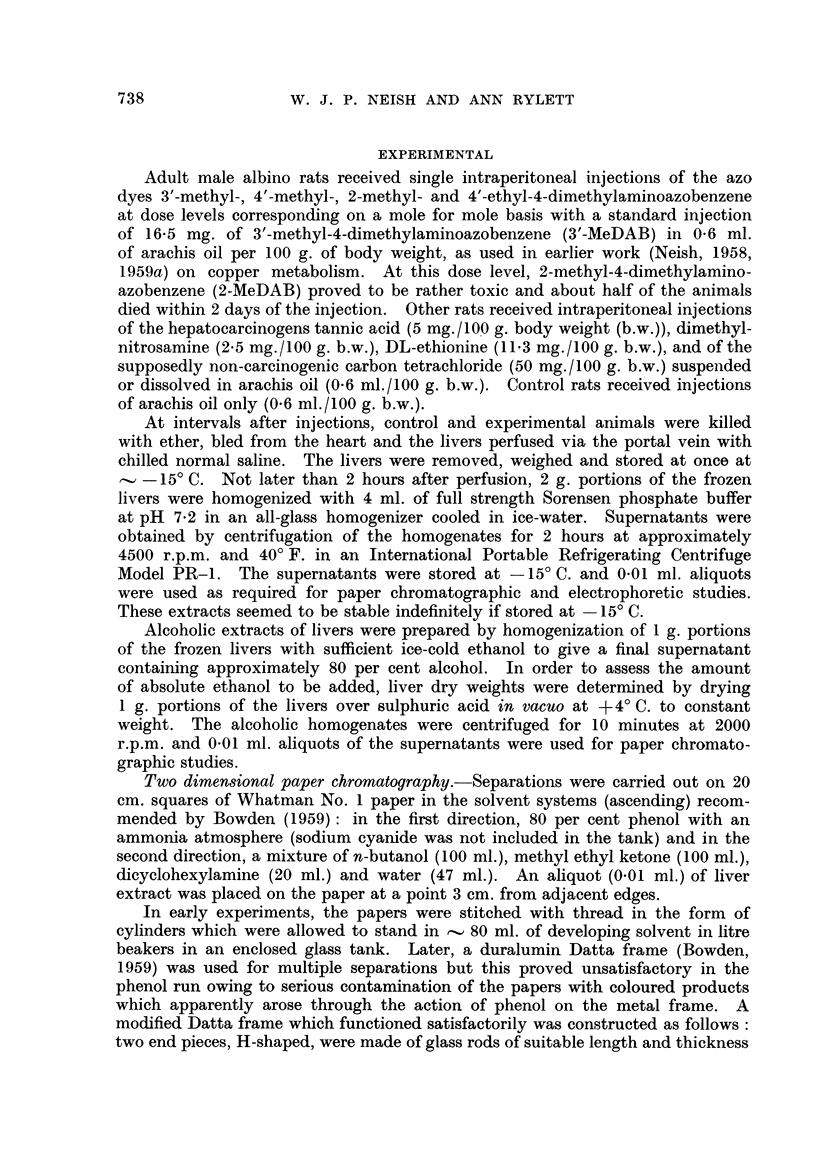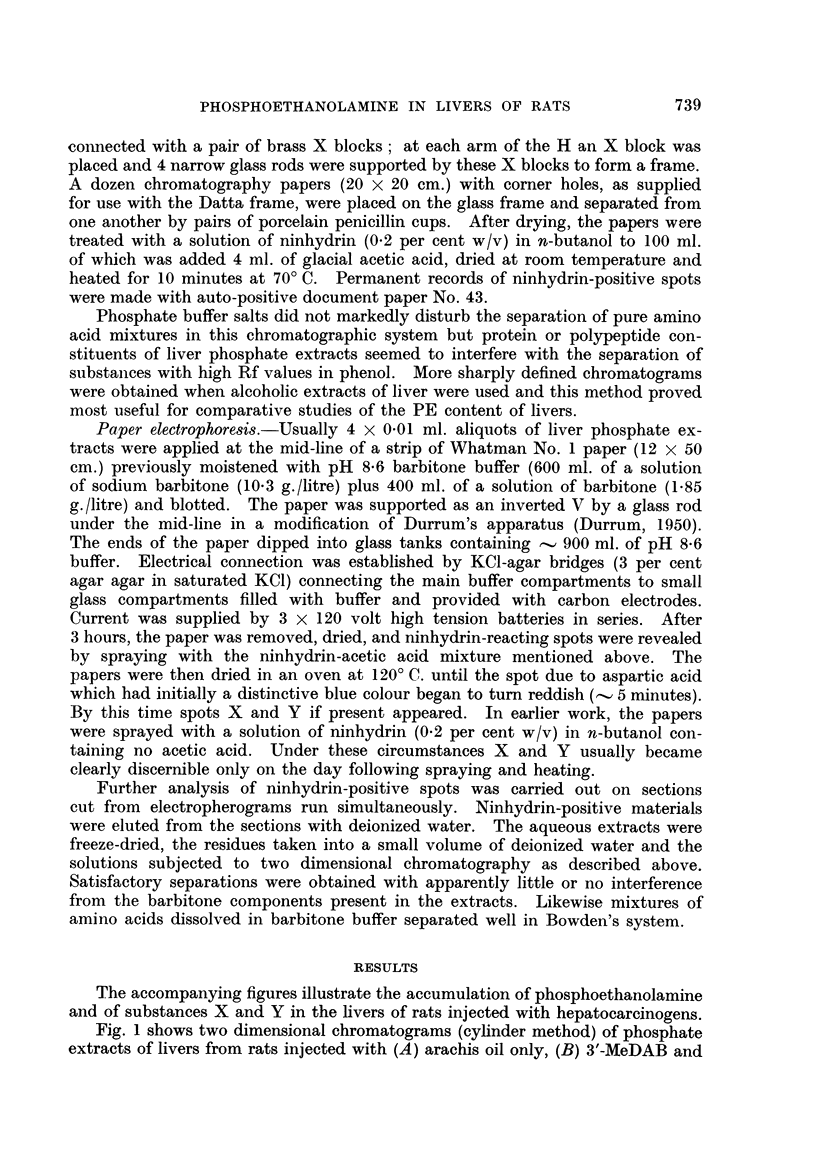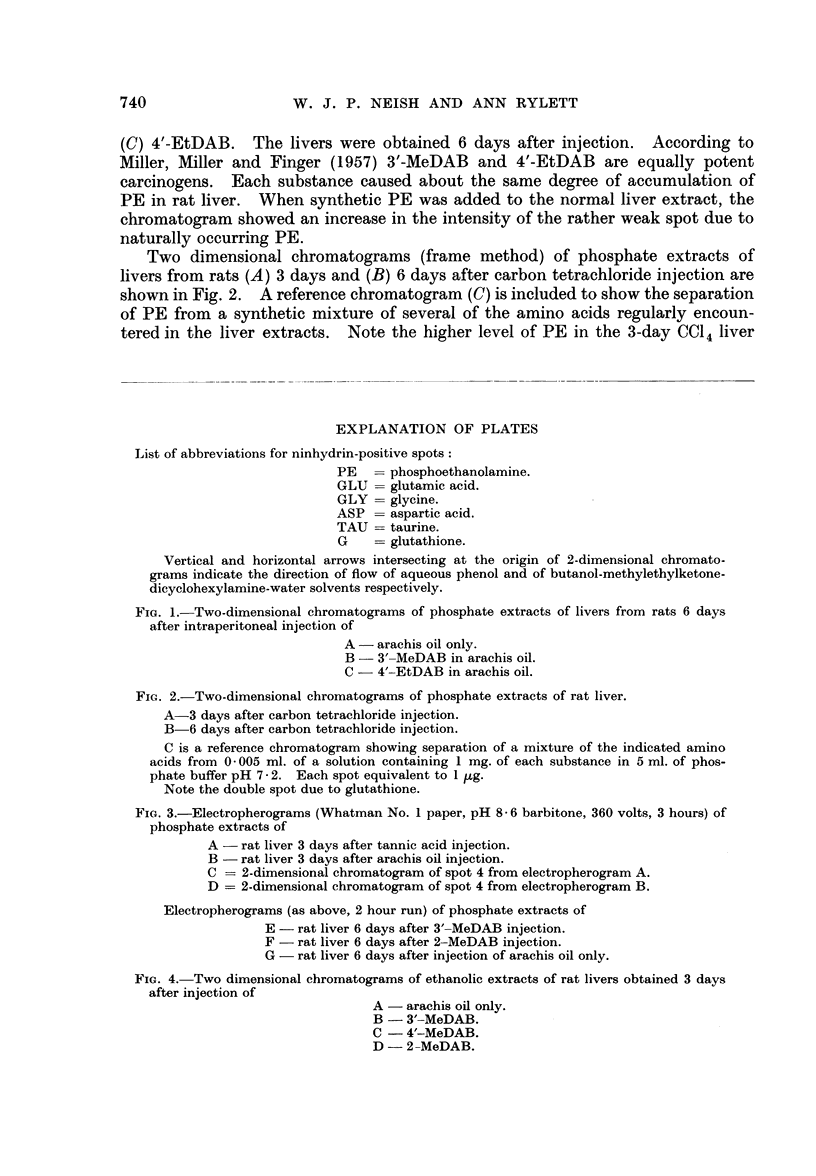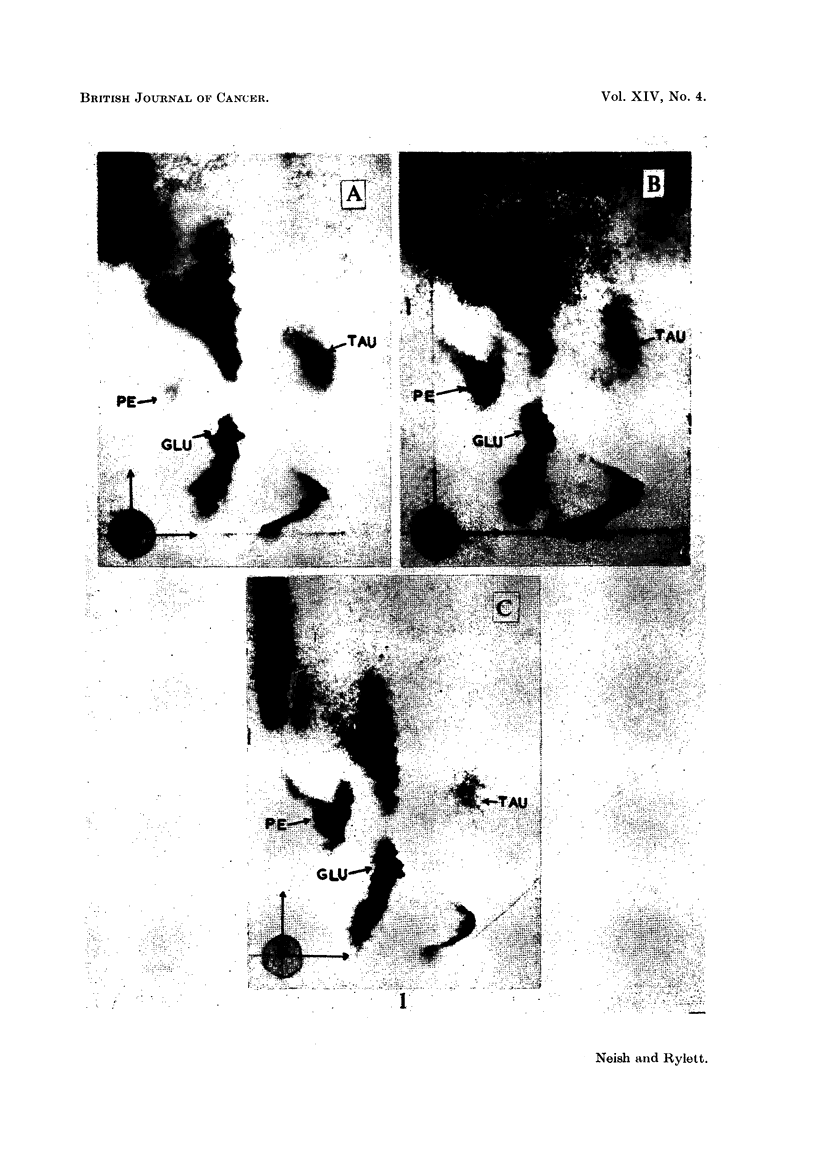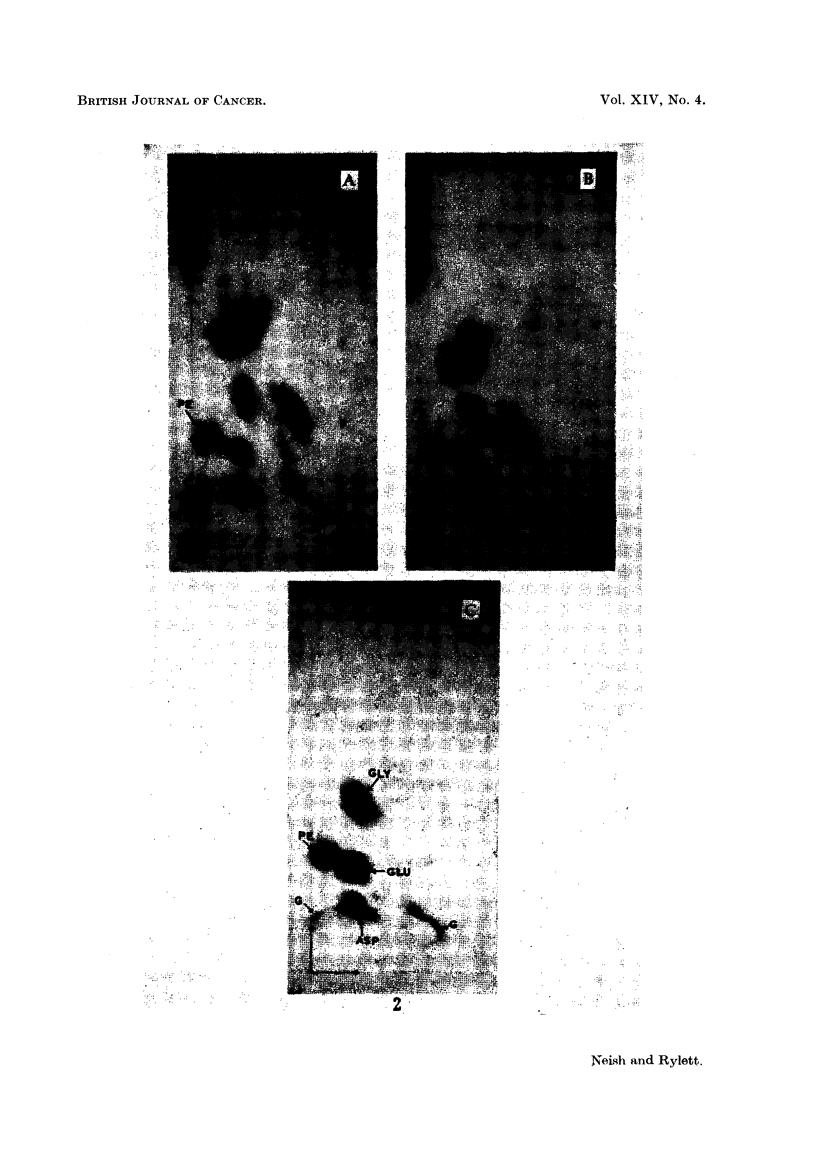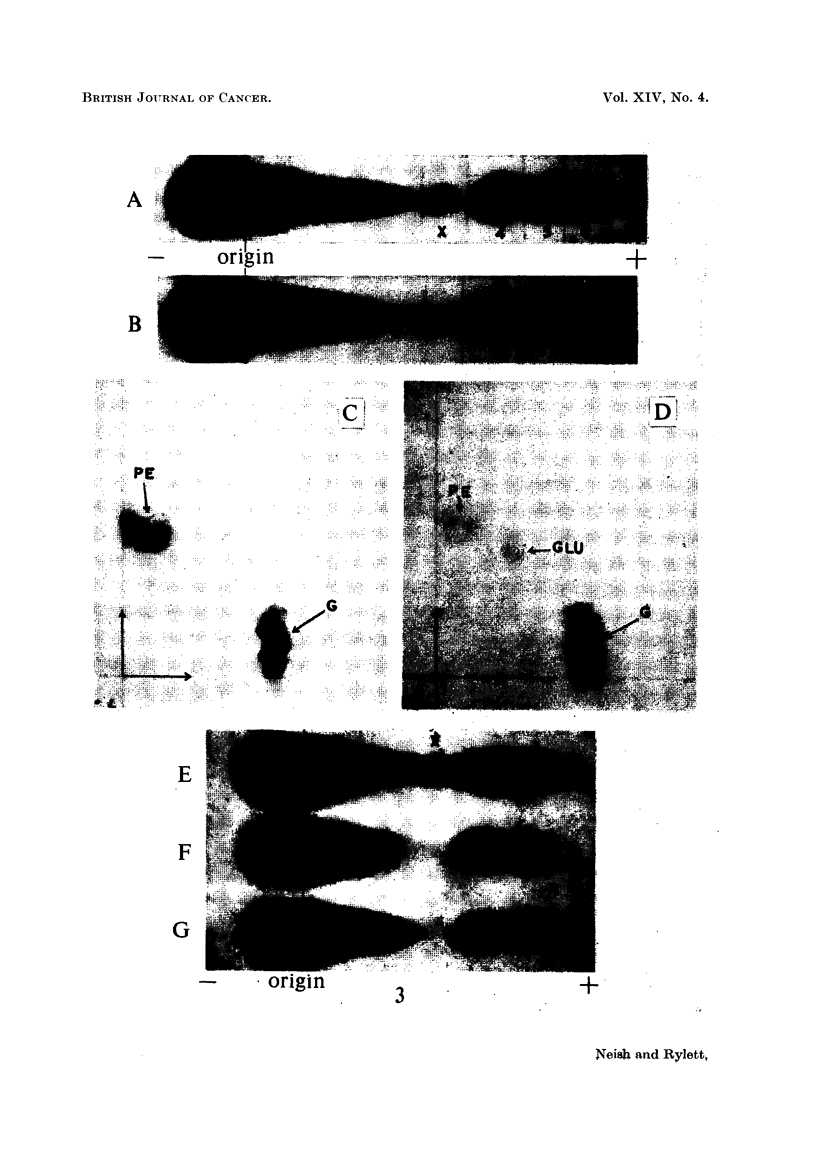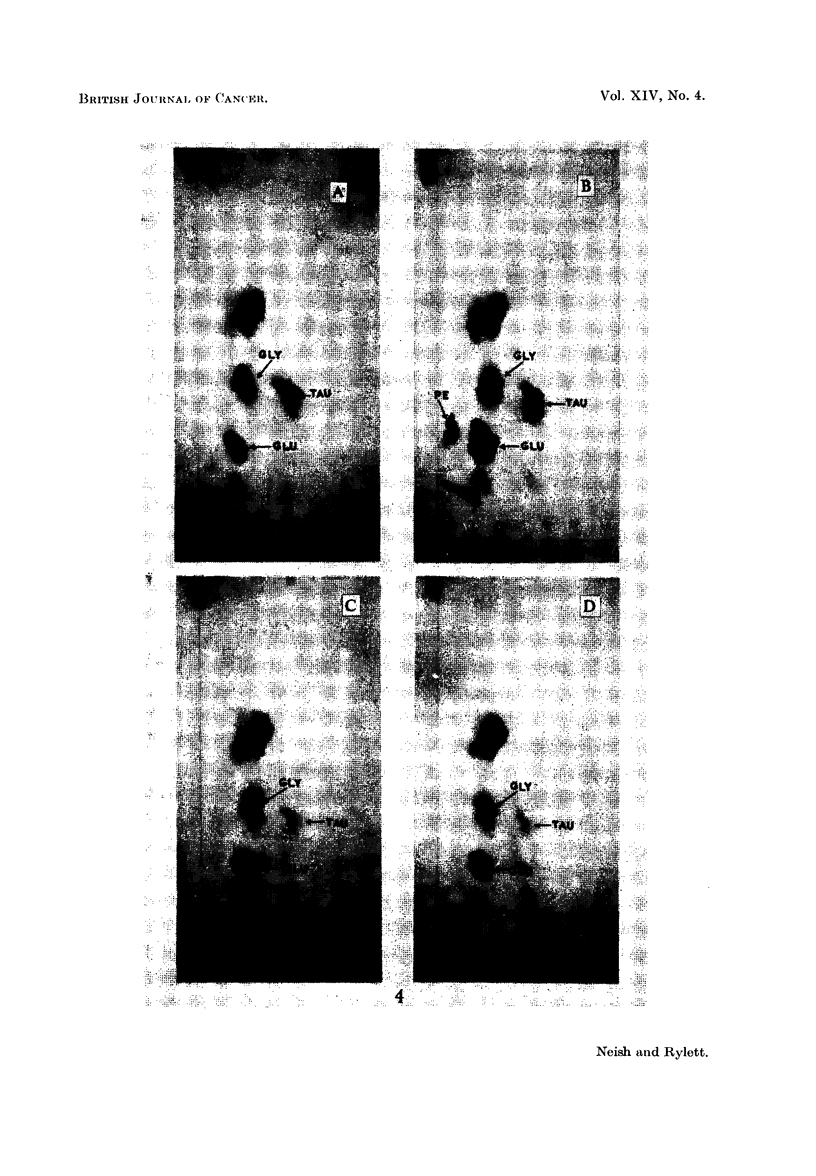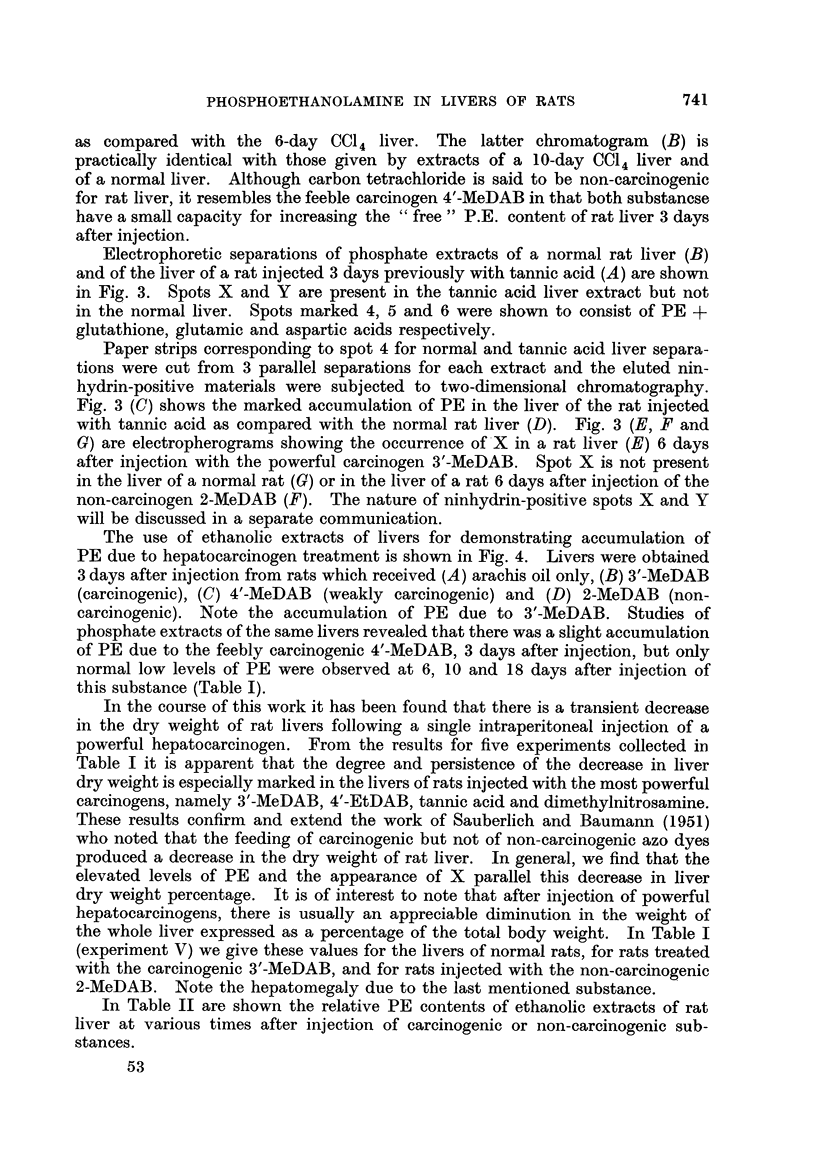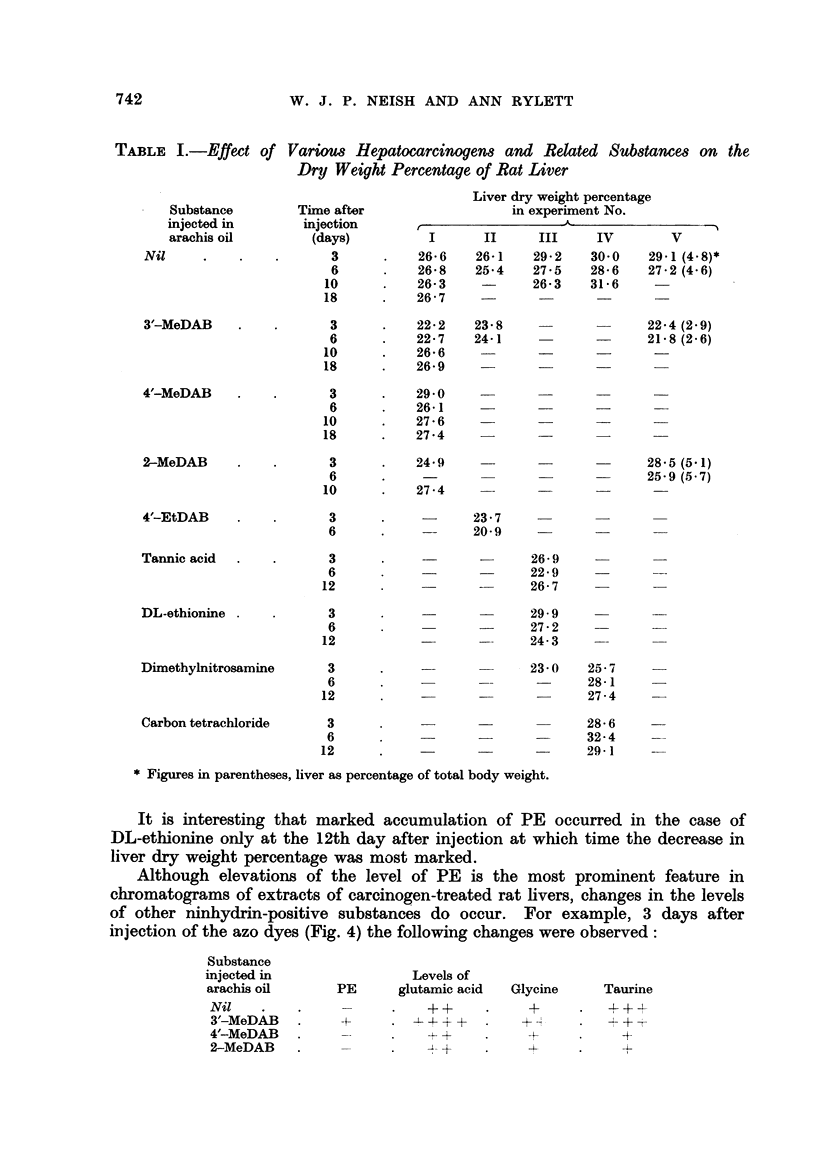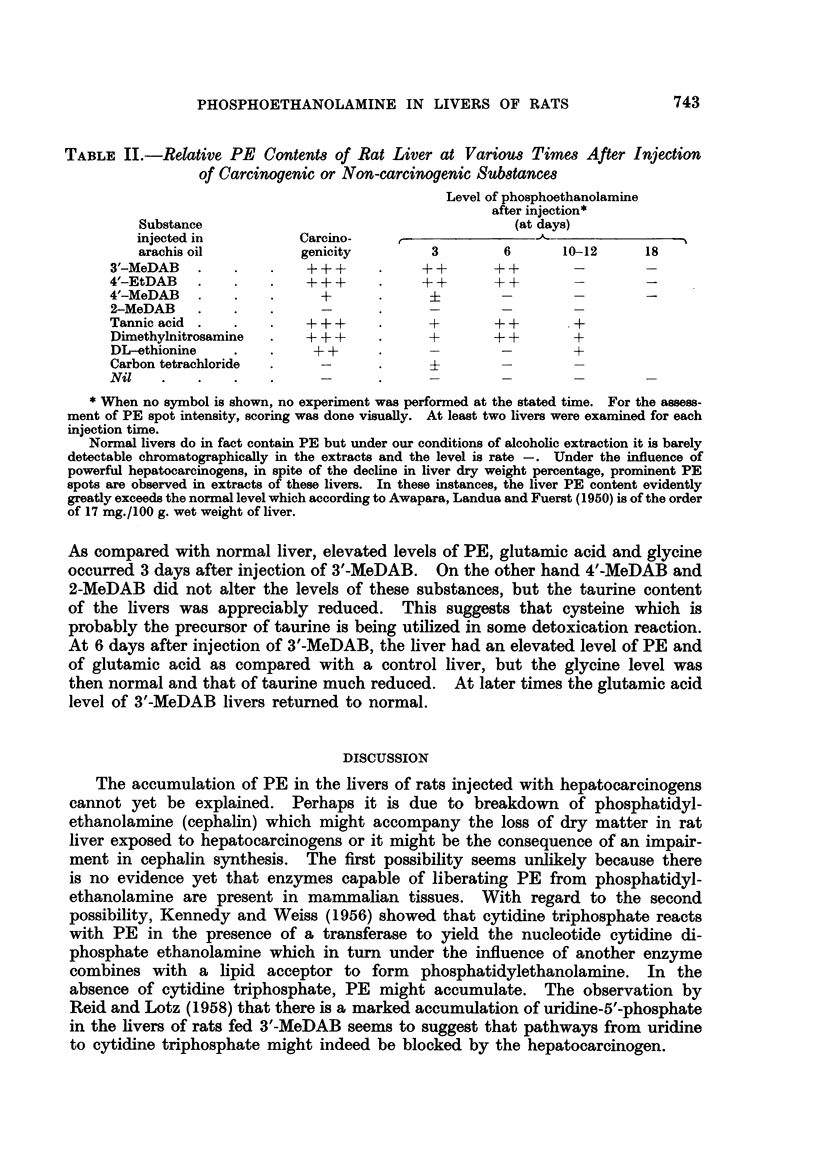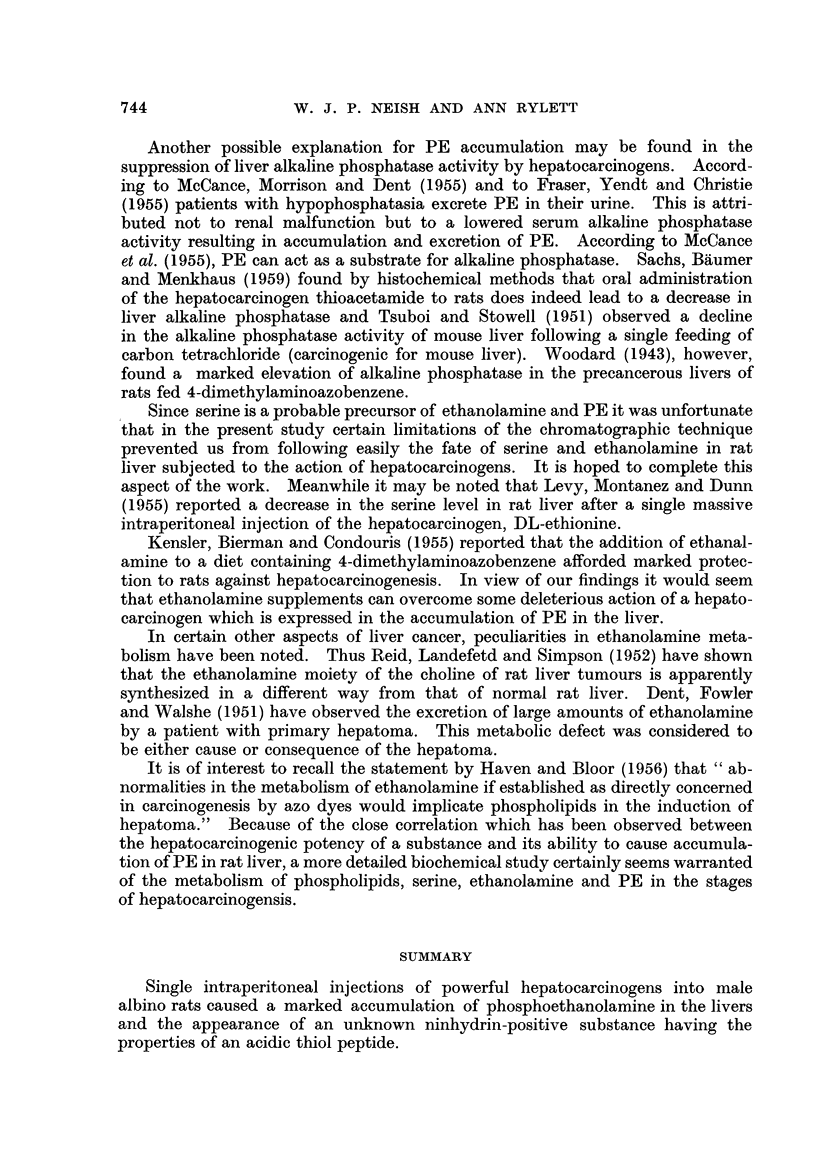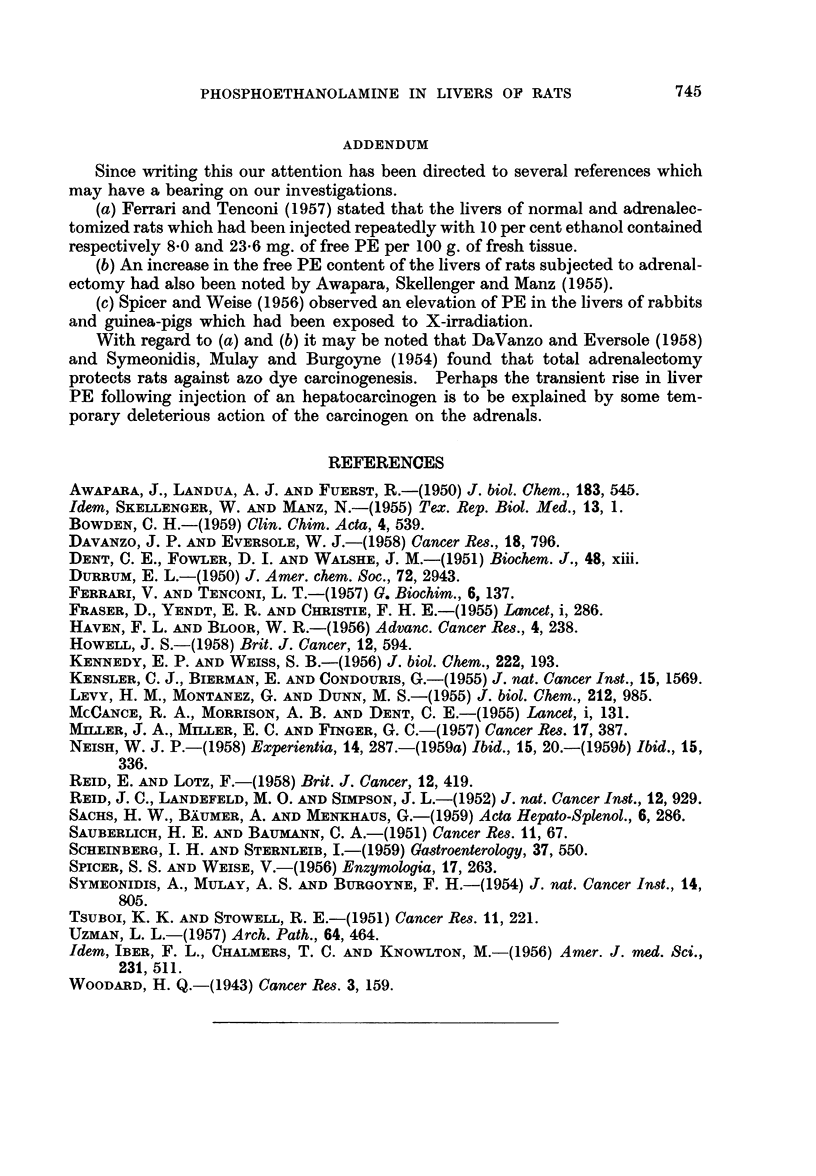# Accumulation of Phosphoethanolamine in the Livers of Rats Injected with Hepatocarcinogens

**DOI:** 10.1038/bjc.1960.84

**Published:** 1960-12

**Authors:** W. J. P. Neish, Ann Rylett

## Abstract

**Images:**


### ACCUMULATION OF PHOSPHOETHANOLAMINE IN THE LIVERS OF RATS INJECTED WITH HEPATOCARCINOGENS

W. J. P. NEISH AND ANN RYLETT

*From the Cancer Research Unit, The University, Sheffield, 10*

Received for publication October 15, 1960

THE addition of copper salts to a diet containing a carcinogenic azo dye affords a degree of protection against hepatocarcinogenesis to rats consuming the diet (Howell, 1958). It has also been found that rats which are fed 3′-methyl-4-dimethylaminoazobenzene (Neish, 1959*b*) or which receive single intraperitoneal injections of various hepatocarcinogens (Neish, 1958, 1959*a*) suffer a marked decline in the serum levels of total copper and of the copper-containing enzyme para-phenylenediamine oxidase (caeruloplasmin). The degree and persistence of this decline appear to be correlated closely with the carcinogenic potency of the injected materials. These results suggest that a derangement of copper metabolism in rat livers may occur at an early stage in hepatocarcinogenesis.

Serum levels of total copper and caeruloplasmin are generally subnormal in patients with hepatolenticular degeneration (Scheinberg and Sternlieb, 1959) and Uzman (1957) and Uzman *et al.* (1956) have detected an abnormal copper-avid ninhydrin-reacting substance, possibly a peptide, in the livers of such patients. It seemed important to determine whether hepatocarcinogens could induce the formation of similar abnormal peptides in rat liver. Although work on this aspect is not yet complete it has been found that injection of hepatocarcinogens into rats increases the levels of certain ninhydrin-positive materials in the liver.

Firstly, it was established qualitatively by paper chromatographic and electrophoretic investigations of phosphate or ethanol extracts of rat liver that intraperitoneal injections of hepatocarcinogens caused a marked increase in the level of "free" phosphoethanolamine (PE) in this tissue. In general, a strong hepatocarcinogen evoked and maintained a higher level of PE for a longer time than did a weaker one.

Secondly, it was found that the livers of rats injected with hepatocarcinogens contained one or two unidentified ninhydrin-positive substances X and Y which migrated towards the anode during paper electrophoresis at pH 8·6. Spot X was observed regularly after the application of a strong hepatocarcinogen and its intensity seemed to run parallel with the intensity of PE. Y was not always present when X occurred but it often appeared when X was rather intense. Traces of X but not of Y have been noted occasionally in extracts of normal rat liver. The nature of X and Y has not yet been completely elucidated but there is evidence that these substances are acidic peptides and that X at least seems to be closely related to glutathione.

In the present paper we shall discuss in detail the effect of hepatocarcinogens on the content of free PE in rat livers. In a future publication the occurrence and nature of the peptide-like materials X and Y will be considered in relation to similar substances which have been found in the livers of tumour-bearing rats and in tumour extracts.

### EXPERIMENTAL

Adult male albino rats received single intraperitoneal injections of the azo dyes 3'-methyl-, 4'-methyl-, 2-methyl- and 4'-ethyl-4-dimethylaminoazobenzene at dose levels corresponding on a mole for mole basis with a standard injection of 16·5 mg. of 3'-methyl-4-dimethylaminoazobenzene (3'-MeDAB) in 0·6 ml. of arachis oil per 100 g. of body weight, as used in earlier work (Neish, 1958, 1959*a*) on copper metabolism. At this dose level, 2-methyl-4-dimethylamino-azobenzene (2-MeDAB) proved to be rather toxic and about half of the animals died within 2 days of the injection. Other rats received intraperitoneal injections of the hepatocarcinogens tannic acid (5 mg./100 g. body weight (b.w.)), dimethyl-nitrosamine (2·5 mg./100 g. b.w.), DL-ethionine (11·3 mg./100 g. b.w.), and of the supposedly non-carcinogenic carbon tetrachloride (50 mg./100 g. b.w.) suspended or dissolved in arachis oil (0·6 ml./100 g. b.w.). Control rats received injections of arachis oil only (0·6 ml./100 g. b.w.).

At intervals after injections, control and experimental animals were killed with ether, bled from the heart and the livers perfused via the portal vein with chilled normal saline. The livers were removed, weighed and stored at once at ~ −15° C. Not later than 2 hours after perfusion, 2 g. portions of the frozen livers were homogenized with 4 ml. of full strength Sorensen phosphate buffer at pH 7·2 in an all-glass homogenizer cooled in ice-water. Supernatants were obtained by centrifugation of the homogenates for 2 hours at approximately 4500 r.p.m. and 40° F. in an International Portable Refrigerating Centrifuge Model PR–1. The supernatants were stored at −15° C. and 0·01 ml. aliquots were used as required for paper chromatographic and electrophoretic studies. These extracts seemed to be stable indefinitely if stored at −15° C.

Alcoholic extracts of livers were prepared by homogenization of 1 g. portions of the frozen livers with sufficient ice-cold ethanol to give a final supernatant containing approximately 80 per cent alcohol. In order to assess the amount of absolute ethanol to be added, liver dry weights were determined by drying 1 g. portions of the livers over sulphuric acid *in vacuo* at +4° C. to constant weight. The alcoholic homogenates were centrifuged for 10 minutes at 2000 r.p.m. and 0·01 ml. aliquots of the supernatants were used for paper chromato-graphic studies.

*Two dimensional paper chromatography.*—Separations were carried out on 20 cm. squares of Whatman No. 1 paper in the solvent systems (ascending) recommended by Bowden (1959): in the first direction, 80 per cent phenol with an ammonia atmosphere (sodium cyanide was not included in the tank) and in the second direction, a mixture of *n*-butanol (100 ml.), methyl ethyl ketone (100 ml.), dicyclohexylamine (20 ml.) and water (47 ml.). An aliquot (0·01 ml.) of liver extract was placed on the paper at a point 3 cm. from adjacent edges.

In early experiments, the papers were stitched with thread in the form of cylinders which were allowed to stand in ~ 80 ml. of developing solvent in litre beakers in an enclosed glass tank. Later, a duralumin Datta frame (Bowden, 1959) was used for multiple separations but this proved unsatisfactory in the phenol run owing to serious contamination of the papers with coloured products which apparently arose through the action of phenol on the metal frame. A modified Datta frame which functioned satisfactorily was constructed as follows: two end pieces, H-shaped, were made of glass rods of suitable length and thickness connected with a pair of brass X blocks ; at each arm of the H an X block was placed and 4 narrow glass rods were supported by these X blocks to form a frame. A dozen chromatography papers (20 × 20 cm.) with corner holes, as supplied for use with the Datta frame, were placed on the glass frame and separated from one another by pairs of porcelain penicillin cups. After drying, the papers were treated with a solution of ninhydrin (0·2 per cent w/v) in *n*-butanol to 100 ml. of which was added 4 ml. of glacial acetic acid, dried at room temperature and heated for 10 minutes at 70° C. Permanent records of ninhydrin-positive spots were made with auto-positive document paper No. 43.

Phosphate buffer salts did not markedly disturb the separation of pure amino acid mixtures in this chromatographic system but protein or polypeptide constituents of liver phosphate extracts seemed to interfere with the separation of substances with high Rf values in phenol. More sharply defined chromatograms were obtained when alcoholic extracts of liver were used and this method proved most useful for comparative studies of the PE content of livers.

*Paper electrophoresis.*—Usually 4 × 0·01 ml. aliquots of liver phosphate extracts were applied at the mid-line of a strip of Whatman No. 1 paper (12 × 50 cm.) previously moistened with pH 8·6 barbitone buffer (600 ml. of a solution of sodium barbitone (10·3 g./litre) plus 400 ml. of a solution of barbitone (1·85 g./litre) and blotted. The paper was supported as an inverted V by a glass rod under the mid-line in a modification of Durrum's apparatus (Durrum, 1950). The ends of the paper dipped into glass tanks containing ~ 900 ml. of pH 8·6 buffer. Electrical connection was established by KCl-agar bridges (3 per cent agar agar in saturated KCl) connecting the main buffer compartments to small glass compartments filled with buffer and provided with carbon electrodes. Current was supplied by 3 × 120 volt high tension batteries in series. After 3 hours, the paper was removed, dried, and ninhydrin-reacting spots were revealed by spraying with the ninhydrin-acetic acid mixture mentioned above. The papers were then dried in an oven at 120° C. until the spot due to aspartic acid which had initially a distinctive blue colour began to turn reddish (~ 5 minutes). By this time spots X and Y if present appeared. In earlier work, the papers were sprayed with a solution of ninhydrin (0·2 per cent w/v) in *n*-butanol containing no acetic acid. Under these circumstances X and Y usually became clearly discernible only on the day following spraying and heating.

Further analysis of ninhydrin-positive spots was carried out on sections cut from electropherograms run simultaneously. Ninhydrin-positive materials were eluted from the sections with deionized water. The aqueous extracts were freeze-dried, the residues taken into a small volume of deionized water and the solutions subjected to two dimensional chromatography as described above. Satisfactory separations were obtained with apparently little or no interference from the barbitone components present in the extracts. Likewise mixtures of amino acids dissolved in barbitone buffer separated well in Bowden's system.

### RESULTS

The accompanying figures illustrate the accumulation of phosphoethanolamine and of substances X and Y in the livers of rats injected with hepatocarcinogens.

Fig. 1 shows two dimensional chromatograms (cylinder method) of phosphate extracts of livers from rats injected with (*A*) arachis oil only, (*B*) 3′-MeDAB and

(*C*) 4′-EtDAB. The livers were obtained 6 days after injection. According to Miller, Miller and Finger (1957) 3′-MeDAB and 4′-EtDAB are equally potent carcinogens. Each substance caused about the same degree of accumulation of PE in rat liver. When synthetic PE was added to the normal liver extract, the chromatogram showed an increase in the intensity of the rather weak spot due to naturally occurring PE.

Two dimensional chromatograms (frame method) of phosphate extracts of livers from rats (*A*) 3 days and (*B*) 6 days after carbon tetrachloride injection are shown in Fig. 2. A reference chromatogram (*C*) is included to show the separation of PE from a synthetic mixture of several of the amino acids regularly encountered in the liver extracts. Note the higher level of PE in the 3-day $CCl_4$ liver

---

EXPLANATION OF PLATES

List of abbreviations for ninhydrin-positive spots :

PE = phosphoethanolamine.
GLU = glutamic acid.
GLY = glycine.
ASP = aspartic acid.
TAU = taurine.
G = glutathione.

Vertical and horizontal arrows intersecting at the origin of 2-dimensional chromatograms indicate the direction of flow of aqueous phenol and of butanol-methylethylketone-dicyclohexylamine-water solvents respectively.

FIG. 1.—Two-dimensional chromatograms of phosphate extracts of livers from rats 6 days after intraperitoneal injection of

A — arachis oil only.
B — 3′-MeDAB in arachis oil.
C — 4′-EtDAB in arachis oil.

FIG. 2.—Two-dimensional chromatograms of phosphate extracts of rat liver.

A—3 days after carbon tetrachloride injection.
B—6 days after carbon tetrachloride injection.

C is a reference chromatogram showing separation of a mixture of the indicated amino acids from 0·005 ml. of a solution containing 1 mg. of each substance in 5 ml. of phosphate buffer pH 7·2. Each spot equivalent to 1 μg.

Note the double spot due to glutathione.

FIG. 3.—Electropherograms (Whatman No. 1 paper, pH 8·6 barbitone, 360 volts, 3 hours) of phosphate extracts of

A — rat liver 3 days after tannic acid injection.
B — rat liver 3 days after arachis oil injection.
C = 2-dimensional chromatogram of spot 4 from electropherogram A.
D = 2-dimensional chromatogram of spot 4 from electropherogram B.

Electropherograms (as above, 2 hour run) of phosphate extracts of

E — rat liver 6 days after 3′-MeDAB injection.
F — rat liver 6 days after 2-MeDAB injection.
G — rat liver 6 days after injection of arachis oil only.

FIG. 4.—Two dimensional chromatograms of ethanolic extracts of rat livers obtained 3 days after injection of

A — arachis oil only.
B — 3′-MeDAB.
C — 4′-MeDAB.
D — 2-MeDAB.

1

Neish and Rylett.

2

3

Neish and Rylett.

4

Neish and Rylett.

as compared with the 6-day $CCl_4$ liver. The latter chromatogram (*B*) is practically identical with those given by extracts of a 10-day $CCl_4$ liver and of a normal liver. Although carbon tetrachloride is said to be non-carcinogenic for rat liver, it resembles the feeble carcinogen 4′-MeDAB in that both substancse have a small capacity for increasing the " free " P.E. content of rat liver 3 days after injection.

Electrophoretic separations of phosphate extracts of a normal rat liver (*B*) and of the liver of a rat injected 3 days previously with tannic acid (*A*) are shown in Fig. 3. Spots X and Y are present in the tannic acid liver extract but not in the normal liver. Spots marked 4, 5 and 6 were shown to consist of PE + glutathione, glutamic and aspartic acids respectively.

Paper strips corresponding to spot 4 for normal and tannic acid liver separations were cut from 3 parallel separations for each extract and the eluted ninhydrin-positive materials were subjected to two-dimensional chromatography. Fig. 3 (*C*) shows the marked accumulation of PE in the liver of the rat injected with tannic acid as compared with the normal rat liver (*D*). Fig. 3 (*E*, *F* and *G*) are electropherograms showing the occurrence of X in a rat liver (*E*) 6 days after injection with the powerful carcinogen 3′-MeDAB. Spot X is not present in the liver of a normal rat (*G*) or in the liver of a rat 6 days after injection of the non-carcinogen 2-MeDAB (*F*). The nature of ninhydrin-positive spots X and Y will be discussed in a separate communication.

The use of ethanolic extracts of livers for demonstrating accumulation of PE due to hepatocarcinogen treatment is shown in Fig. 4. Livers were obtained 3 days after injection from rats which received (*A*) arachis oil only, (*B*) 3′-MeDAB (carcinogenic), (*C*) 4′-MeDAB (weakly carcinogenic) and (*D*) 2-MeDAB (non-carcinogenic). Note the accumulation of PE due to 3′-MeDAB. Studies of phosphate extracts of the same livers revealed that there was a slight accumulation of PE due to the feebly carcinogenic 4′-MeDAB, 3 days after injection, but only normal low levels of PE were observed at 6, 10 and 18 days after injection of this substance (Table I).

In the course of this work it has been found that there is a transient decrease in the dry weight of rat livers following a single intraperitoneal injection of a powerful hepatocarcinogen. From the results for five experiments collected in Table I it is apparent that the degree and persistence of the decrease in liver dry weight is especially marked in the livers of rats injected with the most powerful carcinogens, namely 3′-MeDAB, 4′-EtDAB, tannic acid and dimethylnitrosamine. These results confirm and extend the work of Sauberlich and Baumann (1951) who noted that the feeding of carcinogenic but not of non-carcinogenic azo dyes produced a decrease in the dry weight of rat liver. In general, we find that the elevated levels of PE and the appearance of X parallel this decrease in liver dry weight percentage. It is of interest to note that after injection of powerful hepatocarcinogens, there is usually an appreciable diminution in the weight of the whole liver expressed as a percentage of the total body weight. In Table I (experiment V) we give these values for the livers of normal rats, for rats treated with the carcinogenic 3′-MeDAB, and for rats injected with the non-carcinogenic 2-MeDAB. Note the hepatomegaly due to the last mentioned substance.

In Table II are shown the relative PE contents of ethanolic extracts of rat liver at various times after injection of carcinogenic or non-carcinogenic substances.

TABLE I.—*Effect of Various Hepatocarcinogens and Related Substances on the Dry Weight Percentage of Rat Liver*

| Substance injected in arachis oil | Time after injection (days) | Liver dry weight percentage in experiment No. | | | | |
|---|---|---|---|---|---|---|
| | | I | II | III | IV | V |
| *Nil* | 3 | 26·6 | 26·1 | 29·2 | 30·0 | 29·1 (4·8)* |
| | 6 | 26·8 | 25·4 | 27·5 | 28·6 | 27·2 (4·6) |
| | 10 | 26·3 | — | 26·3 | 31·6 | — |
| | 18 | 26·7 | — | — | — | — |
| 3′-MeDAB | 3 | 22·2 | 23·8 | — | — | 22·4 (2·9) |
| | 6 | 22·7 | 24·1 | — | — | 21·8 (2·6) |
| | 10 | 26·6 | — | — | — | — |
| | 18 | 26·9 | — | — | — | — |
| 4′-MeDAB | 3 | 29·0 | — | — | — | — |
| | 6 | 26·1 | — | — | — | — |
| | 10 | 27·6 | — | — | — | — |
| | 18 | 27·4 | — | — | — | — |
| 2-MeDAB | 3 | 24·9 | — | — | — | 28·5 (5·1) |
| | 6 | — | — | — | — | 25·9 (5·7) |
| | 10 | 27·4 | — | — | — | — |
| 4′-EtDAB | 3 | — | 23·7 | — | — | — |
| | 6 | — | 20·9 | — | — | — |
| Tannic acid | 3 | — | — | 26·9 | — | — |
| | 6 | — | — | 22·9 | — | — |
| | 12 | — | — | 26·7 | — | — |
| DL-ethionine | 3 | — | — | 29·9 | — | — |
| | 6 | — | — | 27·2 | — | — |
| | 12 | — | — | 24·3 | — | — |
| Dimethylnitrosamine | 3 | — | — | 23·0 | 25·7 | — |
| | 6 | — | — | — | 28·1 | — |
| | 12 | — | — | — | 27·4 | — |
| Carbon tetrachloride | 3 | — | — | — | 28·6 | — |
| | 6 | — | — | — | 32·4 | — |
| | 12 | — | — | — | 29·1 | — |

* Figures in parentheses, liver as percentage of total body weight.

It is interesting that marked accumulation of PE occurred in the case of DL-ethionine only at the 12th day after injection at which time the decrease in liver dry weight percentage was most marked.

Although elevations of the level of PE is the most prominent feature in chromatograms of extracts of carcinogen-treated rat livers, changes in the levels of other ninhydrin-positive substances do occur. For example, 3 days after injection of the azo dyes (Fig. 4) the following changes were observed :

| Substance injected in arachis oil | PE | Levels of glutamic acid | Glycine | Taurine |
|---|---|---|---|---|
| *Nil* | — | ++ | + | +++ |
| 3′-MeDAB | + | ++++ | ++ | +++ |
| 4′-MeDAB | — | ++ | + | + |
| 2-MeDAB | — | ++ | + | + |

TABLE II.—*Relative PE Contents of Rat Liver at Various Times After Injection of Carcinogenic or Non-carcinogenic Substances*

| Substance injected in arachis oil | Carcino-genicity | Level of phosphoethanolamine after injection* (at days) 3 | 6 | 10–12 | 18 |
|---|---|---|---|---|---|
| 3′-MeDAB | +++ | ++ | ++ | − | − |
| 4′-EtDAB | +++ | ++ | ++ | − | − |
| 4′-MeDAB | + | ± | − | − | − |
| 2-MeDAB | − | − | − | − | |
| Tannic acid | +++ | + | ++ | + | |
| Dimethylnitrosamine | +++ | + | ++ | + | |
| DL-ethionine | ++ | − | − | + | |
| Carbon tetrachloride | − | ± | − | − | |
| *Nil* | − | − | − | − | − |

* When no symbol is shown, no experiment was performed at the stated time. For the assessment of PE spot intensity, scoring was done visually. At least two livers were examined for each injection time.

Normal livers do in fact contain PE but under our conditions of alcoholic extraction it is barely detectable chromatographically in the extracts and the level is rate —. Under the influence of powerful hepatocarcinogens, in spite of the decline in liver dry weight percentage, prominent PE spots are observed in extracts of these livers. In these instances, the liver PE content evidently greatly exceeds the normal level which according to Awapara, Landua and Fuerst (1950) is of the order of 17 mg./100 g. wet weight of liver.

As compared with normal liver, elevated levels of PE, glutamic acid and glycine occurred 3 days after injection of 3′-MeDAB. On the other hand 4′-MeDAB and 2-MeDAB did not alter the levels of these substances, but the taurine content of the livers was appreciably reduced. This suggests that cysteine which is probably the precursor of taurine is being utilized in some detoxication reaction. At 6 days after injection of 3′-MeDAB, the liver had an elevated level of PE and of glutamic acid as compared with a control liver, but the glycine level was then normal and that of taurine much reduced. At later times the glutamic acid level of 3′-MeDAB livers returned to normal.

### DISCUSSION

The accumulation of PE in the livers of rats injected with hepatocarcinogens cannot yet be explained. Perhaps it is due to breakdown of phosphatidylethanolamine (cephalin) which might accompany the loss of dry matter in rat liver exposed to hepatocarcinogens or it might be the consequence of an impairment in cephalin synthesis. The first possibility seems unlikely because there is no evidence yet that enzymes capable of liberating PE from phosphatidylethanolamine are present in mammalian tissues. With regard to the second possibility, Kennedy and Weiss (1956) showed that cytidine triphosphate reacts with PE in the presence of a transferase to yield the nucleotide cytidine diphosphate ethanolamine which in turn under the influence of another enzyme combines with a lipid acceptor to form phosphatidylethanolamine. In the absence of cytidine triphosphate, PE might accumulate. The observation by Reid and Lotz (1958) that there is a marked accumulation of uridine-5′-phosphate in the livers of rats fed 3′-MeDAB seems to suggest that pathways from uridine to cytidine triphosphate might indeed be blocked by the hepatocarcinogen.

Another possible explanation for PE accumulation may be found in the suppression of liver alkaline phosphatase activity by hepatocarcinogens. According to McCance, Morrison and Dent (1955) and to Fraser, Yendt and Christie (1955) patients with hypophosphatasia excrete PE in their urine. This is attributed not to renal malfunction but to a lowered serum alkaline phosphatase activity resulting in accumulation and excretion of PE. According to McCance *et al.* (1955), PE can act as a substrate for alkaline phosphatase. Sachs, Bäumer and Menkhaus (1959) found by histochemical methods that oral administration of the hepatocarcinogen thioacetamide to rats does indeed lead to a decrease in liver alkaline phosphatase and Tsuboi and Stowell (1951) observed a decline in the alkaline phosphatase activity of mouse liver following a single feeding of carbon tetrachloride (carcinogenic for mouse liver). Woodard (1943), however, found a marked elevation of alkaline phosphatase in the precancerous livers of rats fed 4-dimethylaminoazobenzene.

Since serine is a probable precursor of ethanolamine and PE it was unfortunate that in the present study certain limitations of the chromatographic technique prevented us from following easily the fate of serine and ethanolamine in rat liver subjected to the action of hepatocarcinogens. It is hoped to complete this aspect of the work. Meanwhile it may be noted that Levy, Montanez and Dunn (1955) reported a decrease in the serine level in rat liver after a single massive intraperitoneal injection of the hepatocarcinogen, DL-ethionine.

Kensler, Bierman and Condouris (1955) reported that the addition of ethanalamine to a diet containing 4-dimethylaminoazobenzene afforded marked protection to rats against hepatocarcinogenesis. In view of our findings it would seem that ethanolamine supplements can overcome some deleterious action of a hepatocarcinogen which is expressed in the accumulation of PE in the liver.

In certain other aspects of liver cancer, peculiarities in ethanolamine metabolism have been noted. Thus Reid, Landefetd and Simpson (1952) have shown that the ethanolamine moiety of the choline of rat liver tumours is apparently synthesized in a different way from that of normal rat liver. Dent, Fowler and Walshe (1951) have observed the excretion of large amounts of ethanolamine by a patient with primary hepatoma. This metabolic defect was considered to be either cause or consequence of the hepatoma.

It is of interest to recall the statement by Haven and Bloor (1956) that "abnormalities in the metabolism of ethanolamine if established as directly concerned in carcinogenesis by azo dyes would implicate phospholipids in the induction of hepatoma." Because of the close correlation which has been observed between the hepatocarcinogenic potency of a substance and its ability to cause accumulation of PE in rat liver, a more detailed biochemical study certainly seems warranted of the metabolism of phospholipids, serine, ethanolamine and PE in the stages of hepatocarcinogensis.

### SUMMARY

Single intraperitoneal injections of powerful hepatocarcinogens into male albino rats caused a marked accumulation of phosphoethanolamine in the livers and the appearance of an unknown ninhydrin-positive substance having the properties of an acidic thiol peptide.

ADDENDUM

Since writing this our attention has been directed to several references which may have a bearing on our investigations.

(*a*) Ferrari and Tenconi (1957) stated that the livers of normal and adrenalectomized rats which had been injected repeatedly with 10 per cent ethanol contained respectively 8·0 and 23·6 mg. of free PE per 100 g. of fresh tissue.

(*b*) An increase in the free PE content of the livers of rats subjected to adrenalectomy had also been noted by Awapara, Skellenger and Manz (1955).

(*c*) Spicer and Weise (1956) observed an elevation of PE in the livers of rabbits and guinea-pigs which had been exposed to X-irradiation.

With regard to (*a*) and (*b*) it may be noted that DaVanzo and Eversole (1958) and Symeonidis, Mulay and Burgoyne (1954) found that total adrenalectomy protects rats against azo dye carcinogenesis. Perhaps the transient rise in liver PE following injection of an hepatocarcinogen is to be explained by some temporary deleterious action of the carcinogen on the adrenals.

### REFERENCES

Awapara, J., Landua, A. J. and Fuerst, R.—(1950) *J. biol. Chem.*, **183**, 545.
*Idem*, Skellenger, W. and Manz, N.—(1955) *Tex. Rep. Biol. Med.*, **13**, 1.
Bowden, C. H.—(1959) *Clin. Chim. Acta*, **4**, 539.
DaVanzo, J. P. and Eversole, W. J.—(1958) *Cancer Res.*, **18**, 796.
Dent, C. E., Fowler, D. I. and Walshe, J. M.—(1951) *Biochem. J.*, **48**, xiii.
Durrum, E. L.—(1950) *J. Amer. chem. Soc.*, **72**, 2943.
Ferrari, V. and Tenconi, L. T.—(1957) *G. Biochim.*, **6**, 137.
Fraser, D., Yendt, E. R. and Christie, F. H. E.—(1955) *Lancet*, i, 286.
Haven, F. L. and Bloor, W. R.—(1956) *Advanc. Cancer Res.*, **4**, 238.
Howell, J. S.—(1958) *Brit. J. Cancer*, **12**, 594.
Kennedy, E. P. and Weiss, S. B.—(1956) *J. biol. Chem.*, **222**, 193.
Kensler, C. J., Bierman, E. and Condouris, G.—(1955) *J. nat. Cancer Inst.*, **15**, 1569.
Levy, H. M., Montanez, G. and Dunn, M. S.—(1955) *J. biol. Chem.*, **212**, 985.
McCance, R. A., Morrison, A. B. and Dent, C. E.—(1955) *Lancet*, i, 131.
Miller, J. A., Miller, E. C. and Finger, G. C.—(1957) *Cancer Res.* **17**, 387.
Neish, W. J. P.—(1958) *Experientia*, **14**, 287.—(1959*a*) *Ibid.*, **15**, 20.—(1959*b*) *Ibid.*, **15**, 336.
Reid, E. and Lotz, F.—(1958) *Brit. J. Cancer*, **12**, 419.
Reid, J. C., Landefeld, M. O. and Simpson, J. L.—(1952) *J. nat. Cancer Inst.*, **12**, 929.
Sachs, H. W., Bäumer, A. and Menkhaus, G.—(1959) *Acta Hepato-Splenol.*, **6**, 286.
Sauberlich, H. E. and Baumann, C. A.—(1951) *Cancer Res.* **11**, 67.
Scheinberg, I. H. and Sternleib, I.—(1959) *Gastroenterology*, **37**, 550.
Spicer, S. S. and Weise, V.—(1956) *Enzymologia*, **17**, 263.
Symeonidis, A., Mulay, A. S. and Burgoyne, F. H.—(1954) *J. nat. Cancer Inst.*, **14**, 805.
Tsuboi, K. K. and Stowell, R. E.—(1951) *Cancer Res.* **11**, 221.
Uzman, L. L.—(1957) *Arch. Path.*, **64**, 464.
*Idem*, Iber, F. L., Chalmers, T. C. and Knowlton, M.—(1956) *Amer. J. med. Sci.*, **231**, 511.
Woodard, H. Q.—(1943) *Cancer Res.* **3**, 159.